# The Great Recession, financial strain and self-assessed health in Ireland

**DOI:** 10.1007/s10198-018-1019-6

**Published:** 2018-12-19

**Authors:** Gintare Mazeikaite, Cathal O’Donoghue, Denisa M. Sologon

**Affiliations:** 1 Luxembourg Institute of Socio-Economic Research (LISER), Esch-Sur-Alzette, Luxembourg; 2 Maastricht University/UNU-MERIT, Maastricht, Netherlands; 3 National University of Ireland, Galway, Ireland

**Keywords:** Population health, Economic crisis, Decomposition, Socio-economic factors, EU-SILC, I14, J00

## Abstract

In this paper, we study the effects of the 2008 economic crisis on general health in one of the most severely affected EU economies—Ireland. We examine the relationship between compositional changes in demographic and socio-economic factors, such as education, income, and financial strain, and changes in the prevalence of poor self-assessed health over a 5-year period (2008–2013). We apply a generalised Oaxaca–Blinder decomposition approach for non-linear regression models proposed by Fairlie (1999, 2005). Results show that the increased financial strain explained the largest part of the increase in poor health in the Irish population and different sub-groups. Changes in the economic activity status and population structure also had a significant positive effect. The expansion of education had a significant negative effect, preventing further increases in poor health. Wealthier and better educated individuals experienced larger relative increases in poor health, which led to reduced socio-economic health inequalities.

## Introduction

The Great Recession that started in 2007 has been the most severe economic downturn since the 1930s [76]. The mortgage crisis quickly trickled down to the collapse of the housing prices and loan defaults, causing severe liquidity issues of major banks worldwide and generous bailout packages. Ultimately, the rate of growth of the global output dropped to a negative 2% in 2009, followed by a gradual increase in joblessness, reduced wages and worsened living standards [76]. In Europe, the crisis has been exacerbated by budgetary pressures and austerity programmes, resulting in the retrenchment of public spending on health care and social welfare. A large body of literature that finds adverse effects of unemployment, income shocks and poor living conditions on health, suggests that a health crisis may follow in the medium and long runs. A rise in depression and suicide rates shortly after the start of the 2008 recession provides early evidence of such effects [19, 36]. While many pathways that link economic crisis to changes in health have been proposed, the relative contributions of different factors are largely unknown. Examining the drivers of the observed health changes may provide evidence for future policy responses aimed at safeguarding population health in times of economic crisis.

We address this gap in the literature by examining how structural changes in the economy affected population health in one of the hardest hit countries by the Great Recession—Ireland—over a 5-year period (2008–2013). The global economic recession coupled with the collapse of the property bubble and the banking crisis prompted the Irish government to seek a loan from the International Monetary Fund (IMF, European Commission and European Central Bank), which came with strict austerity policies that put further pressures on the economy [43, 44, 53]. Due to the fall in private and public investment, the employment rate in Ireland dropped from 69% in 2007 to 59.4% in 2010 after being one of the highest in the EU [9]. Wages stagnated, social welfare benefits were reduced, and the expenditure on healthcare fell sharply, facilitating some efficiency gains in the short-run but also increasing user fees and leading to longer waiting times by 2012 [55]. As a result of high level of indebtedness and unfavourable changes in the economy, population share in mortgage arrears of at least 3 months increased nearly fourfold between 2009 and 2013 [17]. While the effect of the 2008 crisis on population health may vary greatly across countries, Ireland is a particularly relevant case to study due to the magnitude of the crisis and the large increase in financial strain that followed the collapse of the housing bubble.

There are various pathways and mechanisms through which the 2008 economic crisis may have led to worsened population health in Ireland. One plausible pathway is through the reduced expenditure on healthcare, which may have compromised the quality of care or made health services less accessible for certain population groups. Second, high levels of unemployment may have had detrimental economic, psychosocial and behavioural effects on health through stress related to job insecurity and propensity to undertake risky behaviours as a coping strategy [42]. Third, a drop in household income due to unemployment or the fall in working wages may have led to worsened living standards and housing conditions [46]. On the other hand, the effects of the crisis on the neighbourhood quality may have been both positive (through reduced noise and pollution) or negative (trough increased crime in the area) [2, 64, 73]. Finally, the crisis may have exacerbated liquidity issues and led to increased financial strain, which has been linked to deterioration of health [65]. It has been argued that the effects of financial strain on health, in particular, mental health, are often overlooked, and should receive more attention in times of economic recession [4, 85].

In this paper, we examine how structural changes in demographic factors, education, economic activity status, income, financial strain, and environmental factors contributed to changes in the prevalence of poor self-assessed health in the first 5 years of the crisis in Ireland (2008–2013). Decomposition approaches that answer similar questions have been used since the work by Kitagawa [45] in the area of demography. We turn to the family of decomposition methods pioneered in economics by Oaxaca [58] and Blinder [12], who proposed a novel approach to quantify the contributions of explanatory factors to the differentials in the mean dependent variable between population groups and over time. Since 1973, the approach has been extended to examine the changes in distributional statistics other than the mean and has been applied to study various outcomes such as income [7, 13, 69] and, to a lesser extent, health [39, 52, 67, 77, 78]. Because of the binary nature of our dependent variable, we use the decomposition method for non-linear regression models proposed by Fairlie [30, 31].

The contribution of this paper is twofold. First, we quantify the importance of structural changes in demographic and socio-economic factors in shaping health changes in an EU country hit by an economic crisis. To our best knowledge, this has not been done so far. Due to the flexibility of the method, we can distinguish between the effects of demographic factors unrelated to the crisis per se (such as ageing) and a number of socio-economic factors that respond to the changes in the economy (such as income). In addition to this, we study the effects of changes in financial strain on health by including measures of financial strain alongside measures of income. The effects of financial strain on health are often overlooked in the health economics literature [4]. Our hypothesis is that these effects may be particularly important given the nature of the 2008 recession.

We find a significant increase in the prevalence of poor self-assessed health in Ireland between 2008 and 2013. On average, changes in the distribution of demographic and socio-economic factors accounted for three quarters of the observed change. The increased financial strain has been the largest contributor in explaining this effect, followed by the changes in economic activity status and demographic characteristics, in particular, population age structure. The expansion of education, on the other hand, has had a significant protective effect on health. Contrary to our expectation, the health of individuals with higher income and educational attainment was more severely affected by the 2008 crisis in relative terms, which contributed to decreasing income- and education-related health inequalities. To sum up, we find that the increase in the prevalence of poor self-assessed health in Ireland can be largely attributed to the negative changes in socio-economic factors, in particular, the increase in financial strain, which followed the collapse of the housing bubble.

This paper is organised as follows. In the next section, we review the existing literature on the pathways through which economic crises may shape population health. Then we examine the changes in the economy and health indicators in Ireland during the period of 2008–2013. After reviewing the literature and the development of economic and health indicators, we present the methods and data used in this paper. Finally, we review the descriptive and decomposition results and close the paper with a discussion and conclusion.

## Pathways from economic crises to population health: theory and evidence

Although the Great Recession is unique due to its global impact and its complex interactions with national financial and social systems, it made an impact on people’s lives through familiar pathways, such as widespread unemployment and reduced household income. Negative changes in these factors, in turn, are likely to alter material and psychosocial circumstances that are important for preserving good health [23].

Material circumstances comprise consumption potential for essential items such as food and clothing, and physical environment such as housing and neighbourhood quality [46, 50, 68]. For example, reduced income or increased financial uncertainty during an economic crisis may promote consumption of unhealthy foods which are often cheaper than the healthier alternatives, or encourage families to turn down heating to save money [3, 60]. It has been argued that in high-income countries, psychosocial circumstances play a bigger role than material conditions on people’s health [82]. Factors such as negative life events, unemployment and financial indebtedness may produce chronic stress that is linked to reduced immune system response and increased risk of cardiovascular diseases [82]. In addition to this, prolonged exposure to stress may affect health indirectly through the adoption of health-damaging behaviours, such as drinking, smoking or eating for comfort, in particular, among the individuals with fewer socio-economic resources [65, 75].

While there is a large body of literature linking income to health, insufficient attention has been given to the relationship between financial strain and health [4]. A link has been found between debt and a number of health outcomes, in particular, depression and common mental health disorders [21, 54]. Long-term unsecured debt and financial strain have been linked to depressive symptoms in the general population and a health decline among older adults [21, 35, 41]. Interestingly, a study by Zimmerman and Katon [85] that included indicators of income, unemployment and financial strain, found that financial strain was causally related to depression, while income was not. Given that there might be different mechanisms through which the two factors may affect health, it is important to account for both income and financial strain to better understand their causal links to health.

Studies on the Great Depression of the 1920s suggest that it takes 5–7 years for an economic crisis to show its full impact on health [71]. However, some short- and medium-term results already confirm that the recent crisis has taken its toll on population health in Europe and elsewhere. Unfavourable trends associated with the recession have been found in a variety of health measures such as suicide rates, mental health issues, outbreaks of infectious diseases and self-assessed health [1, 19, 43]. On the other hand, a review of past experiences suggests that there might be some positive effects operating at population level, such as decreased traffic fatalities and reduced alcohol consumption [72]. We might expect to find non-uniform effects of the recent crisis on health across different countries and socio-economic groups due to complex interactions between economic opportunities and the degree of protection offered by the welfare state, as suggested by numerous studies that find mitigating effects of social welfare spending and active labour market policies on health [32, 47, 71]. For example, Barroso et al. [8] found increasing education-related health inequalities in Spain during the 2008 economic crisis, whereas the work of Bacigalupe and Escolar-Pujolar [5] suggests that this has not always been the case during economic crises.

To gain an understanding of the ways in which the Great Recession has brought about changes in population health in Ireland, the recent changes in health and socio-economic factors in the period of 2008–2013 are discussed in the next section.

## Trends in health and socio-economic factors in Ireland in 2008–2013

After a period of sustained economic growth at an average of 5% between 2001 and 2007, the Irish economy saw a sharp decline in national output in 2008 (Table 1). One of the most affected sectors was the construction sector, which saw the share of the value-added fall from 6.6% in 2008 to 1.7% in 2010. After encountering a growing budget deficit, the Irish government accepted a rescue package from the IMF, EU and European Central Bank, which put further pressure on the economy. The rapid decline in economic activity quickly affected the Irish labour market, with the unemployment rate doubling from 6.4 to 12% between 2008 and 2009, and peaking at 14.7% in 2012. Men were affected somewhat more than women in absolute, but not relative terms, and at its peak in 2012, the youth unemployment rate was the highest among the Western European OECD countries. One of the most pronounced changes occurred in long-term unemployment, which rose from 26.2% in 2008 to an alarming 61.7% in 2012. Long-term unemployment may be a source of poor health both due to the psychosocial effects (stigma and loss of social status related to being long-term unemployed or being a welfare recipient) and due to withdrawal of unemployment benefits [14, 70].

**Table 1:** Economic and social indicators

Year	2008	2009	2010	2011	2012	2013	Change, % 2008/2013
National accounts$$^{\text {a}}$$							
GDP per capita, $ PPPs	44200.5	41532.7	43225.1	45473.4	46519.4	48316.6	9.3
Govt deficit, % GDP	− 7.0	$$-$$13.8	− 32.1	$$-$$12.6	− 8.0	$$-$$5.7	− 3.7
Share of real value added, %$$^{\text {a}}$$							
Construction	6.6	2.7	1.7	1.7	2.3	2.8	− 57.6
Finance and insurance	9.8	10.5	11.3	9.6	9.1	8.0	− 18.4
Average price of a house, thousand EUR$$^{\text {b}}$$							
New	305.3	242.0	228.3	230.3	220.4	228.2	− 25.3
Second hand	348.8	275.3	274.1	260.4	249.1	255.9	− 26.6
Unemployment rate, % of labour force$$^{\text {a}}$$							
Total	6.4	12.0	13.9	14.6	14.7	13.0	103.1
Males	7.5	15.0	17.0	17.7	17.7	15.0	100.0
Females	4.9	8.2	9.8	10.8	11.0	10.7	118.4
Youth (ages 15–24)	13.3$$^{\upbeta}$$	24.0	27.6	29.0	30.4	26.8	101.5
Long-term unemployment, %$$^{\text {a}}$$							
In total unemployed	26.5	29.1	49.1	59.3	61.7	60.6	128.7
Loan accounts in arrears for more than 90 days, %$$^{\text {c}}$$							
In all arrears	–	3.6	5.7	9.0	11.9	12.6	250.0$$^\upbeta$$

Sources: $^{\text {a}}$OECD [63], $^{\text {b}}$CSO [25], $^{\text {c}}$Central Bank of Ireland [18]

^β^Break in series (nearest estimate used)

Another important change in the economy was the fall of the housing prices, which created difficulties for homeowners, especially those with debt obligations. Faced with reduced income due to joblessness or shorter working hours, an increasing number of people were unable to meet their loan obligations, as shown by a more than twofold rise in arrears for above 90 days between 2008 and 2013. The rates of increase are alarming for health: individuals with mortgage arrears are shown to be at risk of mental health problems, deteriorating self-assessed health and more frequent GP visits [11, 20, 56], while loss of home is linked to the increased risk of suicide [34].

Between 2008 and 2009, the Irish government adopted countercyclical policies regarding healthcare expenditure [74]. Despite the pressures on the public budget, healthcare expenditure as a share of GDP continued to rise between 2008 and 2009 (Table 2). One of the countercyclical policies employed by the Irish government centred on medical cards, which enable low-income individuals to get free access to healthcare. In the period of 2008–2013, the percentage of Irish population covered with free medical cards rose from 30.1 to 40.3% [26]. However, after 2009, the Irish government started undertaking a restructuring process of the hospital sector. The reform included a reduction of salaries for health professionals, reduced payments for healthcare providers and policies aimed at curtailing prices of pharmaceutical goods [43, 55]. In addition to this, hospital employment has been reduced, and the number of acute hospital beds continued to decline despite being much below the EU15 average of 402 beds in 2008 [81]. Some burden of healthcare financing has been shifted from the public to the private sector, with increasing out-of-pocket payments due to the rise in user fees, lower reimbursement of dental care for some population groups and the removal of statutory coverage of primary care for wealthy individuals over 70 years of age [55]. The increase in the user payments is particularly alarming for individuals with just above the financial threshold to obtain a medical card [57]. It has been suggested that the reform of the healthcare sector has helped increase its efficiency in the short run, but the rising number of patients on waiting lists and the growing demand for emergency care admissions indicated system deficiencies by 2013 [15].

**Table 2:** Healthcare indicators

	Change, % 2003/2008	2008	2009	2010	2011	2012	2013	Change, % 2008/2013
Health expenditure$^{\text {a}}$								
Total (% of GDP)	22.6	8.6	9.5	8.8	8.1	8.3	8.0	− 7.0
Public sector (% of total)	− 1.7	75.4	72.6	69.6	67.8	67.6	66.6	− 11.7
Private (out-of-pocket) (% of total)	0.1	15.3	16.1	18.2	17.7	16.9	17.4	13.7
Population coverage, %$^{\text {b}}$								
Medical cards	4.5$^{\upbeta}$	30.1	32.6	35.5	37.0	40.4	40.3	33.9
GP visit cards	58.3$^{\upbeta}$	1.9	2.2	2.6	2.7	2.9	2.7	42.1
Healthcare resources$^{\text {a}}$								
Hospital empl. in FTE per 100,000	5.0	1183	1158	1132	1089	1067	1057	− 10.6
Bed occupancy rate (acute care), %	4.2	88.8	89.2	91.4	91.9	92.6	93.8	5.6
Acute care hosp. beds per 100,000	− 8.7	256.6	263.6	256.0	243.0	237.4	239.6	− 6.6

*FTE* full-time equivalent

Sources: $^{\text {a}}$WHO Regional Office for Europe [81]; $^{\text {b}}$Department of Health [26]. $^{\upbeta}$Break in series (nearest estimate in year 2006)

Early signs of the Great Recession in Ireland could be observed with respect to some health indicators, in particular, mental health. As expected, the trend in life expectancy has been fairly stable between 2008 and 2013, but the prevalence of depressive symptoms and rates of suicides and self-inflicted injuries have shown an increase (Table 3). The standardised death rates for mental and behavioural disorders have increased from 7.8 cases per 100,000 population in 2008 to 19.6 per 100,000 population in 2013 [81]. In addition to this, there has been a decrease in the rate of traffic deaths, as observed in previous studies that examined the effects of economic crisis on health [71]. Quite unexpectedly, there has been a decrease in tobacco and alcohol consumption, which might reflect the continuation of the pre-crisis trend or suggest that increased pressures on the household budget may have had a bigger effect than the higher propensity to undertake risky behaviours in times of stress.

**Table 3:** Health and health behaviours

	Change, % 2003/2008	2008	2009	2010	2011	2012	2013	Change, % 2008/2013
Life expectancy$^{\text {a}}$								
At birth (years)	2.2	80.0	80.0	80.8	80.9	80.9	81.1	1.4
Age-standardised death rates (SDR) per 100,000$^{\text {a}}$								
Suicide/self-inflicted injury	0.8	11.3	11.6	10.9	12.1	11.9	10.7	− 5.3
Mental disorders	− 26.1	7.8	10.6	11.8	14.0	16.4	19.6	151.3
Transport accidents	− 24.0	5.7	5.9	4.1	4.1	3.5	3.7	− 35.1
Consumption of substance age 15 + (litres/grammes per capita)$^{\text {b}}$								
Alcohol	− 9.6	12.2	11.0	11.6	11.7	11.5	10.6	− 13.1
Tobacco	− 30.7	1439	1360	1226	1241	1163	1026	− 28.7

Sources: $^{\text {a}}$WHO Regional Office for Europe [81]; $^{\text {b}}$OECD [63]

Overall, the period of economic recession in Ireland has been characterised by worsening socio-economic conditions as well as negative trends in many health indicators, which suggests that changes in socio-economic factors may have mediated the effects of the crisis on health. However, it has been argued that some of the commonly used measures of economic hardship, such as income, may not be able to capture the full impact of the crisis, through, for example, the rise of financial strain [80]. Given the evidence on the effects of material deprivation and psychosocial stress on health, it is important to consider both sets of factors when examining the health impact of the recession. The next chapter discusses the data and the methodological framework that is used to determine the relative importance of the changes in the examined factors in shaping changes in self-assessed population health in Ireland between 2008 and 2013.

## Data and methodology

To study the ways in which the economic recession of 2008 has affected population health in Ireland via various mechanisms, we use data from the European Union Statistics on Income and Living Conditions (EU-SILC). EU-SILC provides annual population representative^1^ information on self-assessed health and a number of demographic and socio-economic variables regarding EU countries. As the effects of the crisis on the labour market in Ireland manifested themselves in 2009 when the unemployment rate doubled compared to 2008, the year 2008 is considered as the ‘pre-crisis’ year and the year 2013 is considered as the ‘crisis’ year. The measure of health we use in our analysis is self-assessed health, which has been shown to be a reliable indicator of morbidity and mortality [37, 40, 48]. The assessment of health consists of one question: “How is your health in general?” and comes with five answer categories from ‘very good’ to ‘very bad’. We identify all answers of less than good health (response categories ‘fair’, ‘bad’ and ‘very bad’) as indicators of poor health. This distinction has also been used in previous studies [16, 49]. The survey provides information on health concerning individuals of 16 years of age and older. We consider five sets of variables for the decomposition:

- Demographics—three variables: age;^2^ gender; marital status (two response categories).
- Education—one variable with three response categories (tertiary, upper secondary, lower than upper secondary).
- Labour market status—one variable with six response categories (employed full-time, employed part-time, unemployed, student, retired, inactive).^3^
- Income—one continuous variable: equivalised household disposable income (square root equivalence scale), 2008 prices.
- Financial strain—six variables with two response categories: difficulty to make ends meet; incapacity to face unexpected expenses; heavy financial burden of the total housing cost; arrears on utility bills; arrears on mortgage or rent payments; arrears on hire purchase instalments/loan payments; heavy financial burden of the repayment of debts.
- Environmental factors—five variables with two response categories: too dark in the dwelling; leaking roof, damp walls/floors; outside noise; pollution, grime and other environmental problems; crime violence or vandalism in the area.

Only individuals who provided full information on the dependent and explanatory variables were included in the estimation sample. Applying this limitation leaves us with a total of 17,955 observations.

For descriptive purposes, we calculated two composite indices: one that combined variables of financial strain and one that combined variables of environmental factors. These two composite indices were used in descriptive statistics to indicate the extent of the change in the prevalence of financial strain and environmental issues in the Irish population in the period of 2008–2013. The composite indicators could be constructed by simply aggregating the number of deprivations or using weights. We use the approach proposed by Desai and Shah [27] and applied by Whelan et al. [80], whereby the variables are weighted by their prevalence in the population, i.e. the more prevalent factors obtain lower weights. The approach relies on the idea that the importance of a deprivation is higher when the person it affects is in a minority as opposed to when the majority of the population has the same deprivation. This approach also adjusts for the decisions made on the deprivation cut-off point of the ordinal variables, such as the difficulty in making ends meet. The described approach can be formalised as follows:

$$\begin{aligned} D_{i} = \sum _{j=1}^{n}(1-p_j) \times k_j, \end{aligned}$$

where $p_j$ is the prevalence of each factor $k_j$ in the pooled estimation sample. The obtained index needs to be standardised and the second cut-off point needs to be chosen to define when an individual is deprived. We consider an individual to be deprived if their deprivation score is higher than that of 75% of the population in 2008:

$$\begin{aligned} I_{D_{ij}} = D_i> p75_{2008}(D_i). \end{aligned}$$

The final deprivation indicator $D_i$ takes values 0 and 1, where 1 denotes a deprivation.

The research question of this study demands a methodology capable of separating the contributions of compositional changes in the examined factors to changes in health. Such methods rely on the seminal work of Oaxaca [58] and Blinder [12] (henceforth OB), who proposed a way to decompose the difference in the mean-dependent variable between two groups or time periods into the contributions of underlying variables. The approach can be described as follows. In a linear regression framework, health at a time *t* could be determined by the following function:

$$\begin{aligned} H_i^t = \beta _0^{t} + \sum _{k=1}^K{\beta _k^t}X_{ik}+\epsilon _i^t, \end{aligned}$$

where *H* is an indicator of poor self-assessed health (1 for poor health and 0 for otherwise), *t* is the year, *X* is a vector of explanatory factors from 1 to *K* and $\epsilon$ is the idiosyncratic error term. Under the zero conditional mean assumption ($E[\epsilon _i^{t}\mid X_i] = 0$) or a weaker ignorability assumption ($\epsilon \perp T\mid X$), the change in population health between 2 years could be expressed as

1 $$\begin{aligned}&\overline{\mathrm{H}}^{t+1} - \overline{\mathrm{H}}^{t} = (\beta _0^{t+1} - \beta _0^{t}) + \overline{\mathrm{X}}_k^{t+1}(\beta _k^{t+1} - \beta _k^{t}) \end{aligned}$$

2 $$\begin{aligned}&+\ (\overline{\mathrm{X}}_k^{t+1} - \overline{\mathrm{X}}_k^{t}).\beta _k^{t}. \end{aligned}$$

Equation 2 would be the part of the overall gap $\overline{\mathrm{H}}^{t+1} - \overline{\mathrm{H}}^{t}$ accounted for by the compositional changes in the vector of explanatory factors $X_k$ and Eq. 1 would encompass the unexplained differences in population health. However, a problem arises when the dependent variable is binary, which makes the OLS regression unsuitable. In the case of a limited dependent variable, the conditional expectation of health would be $E(H \mid T=t) \ne F(E[X \mid T=t];\beta ^t)$, which means that the contributions of the compositional changes in the explanatory variables to the change in health cannot be estimated using the standard OB framework.

Several non-linear decomposition approaches have been proposed in the literature (see for instance Yun [84], Fairlie [30, 31] and Bauer and Sinning [10]). For example, Yun [83] introduced a method to separate the composition and returns structure effects into the contributions of separate covariates, an approach which is independent of the order of decomposition. While path independence in a detailed decomposition is desirable, the results obtained using Yun’s approach may produce inflated estimates if the group differences in some of the covariates are large [29]. In this study, we use the decomposition approach by Fairlie [30, 31]. There is a way to decompose the overall difference in the mean-dependent variable between two periods into the composition effect linked to changes in covariates between 2 years (Eq. 3) and other (Eq. 4) effects in the following way:

3 $$\begin{aligned}&\overline{H}^{t+1} - \overline{H}^{t} = \left[ \sum _{i=1}^{N^{t+1}}{\dfrac{F(X_i^{t+1}\hat{\beta }^{t+1})}{N^{t+1}}} - \sum _{i=1}^{N^{t}}{\dfrac{F(X_i^{t}\hat{\beta }^{t+1})}{N^{t}}}\right] \end{aligned}$$

4 $$\begin{aligned}&+\ \left[ \sum _{i=1}^{N^{t}}{\dfrac{F(X_i^{t}\hat{\beta }^{t+1})}{N^{t}}} - \sum _{i=1}^{N^{t}}{\dfrac{F(X_i^{t}\hat{\beta }^{t})}{N^{t}}}\right] , \end{aligned}$$

where *N* stands for sample sizes in years 2008 and 2013. Alternatively, the counterfactual (the second and third terms in the equation) can be constructed by importing the coefficients $\hat{\beta ^t}$ from the year 2008 into the distribution of covariates observed in the year 2013. The results might differ if the health returns to the explanatory factors vary across 2 years, an index problem that also occurs with the linear OB decomposition. An alternative approach suggested by Oaxaca and Ransom [59] is to weight the terms in the decomposition equation by the coefficients from a pooled sample of two groups $\hat{\beta ^*}$. This approach is applied in the current study.^4^

While the estimation of the aggregate effect is relatively easy to implement, a complication arises when the separate contributions of the covariates *X* to the composition effect are of interest. Fairlie [31] proposed an approach that relies on a one-to-one matching of the observations from both groups, whereby the observations in both samples are ranked by the conditional probabilities estimated using the pooled regression coefficients. Then the effect of covariate $x_1$ could be estimated by importing only this covariate from one distribution to another, keeping other factors constant in the following manner:

5 $$\begin{aligned} \dfrac{1}{N^{t+1}}\sum _{i=1}^{N^{t+1}}{F(\hat{\beta }_{0}^*+X_{1i}^{t+1}\hat{\beta }_{1}^*+ X_{2i}^{t+1}\hat{\beta }_{2}^* ) - F(\hat{\beta }_{0}^*+X_{1i}^{t}\hat{\beta }_{1}^*+ X_{2i}^{t+1}\hat{\beta }_{2}^* ) }. \end{aligned}$$

Several complications may arise in the matching and estimation process described above. First, the number of observations in the two samples may not necessarily be equal, $N^{t+1} \ne N^{t}$. Second, a one-to-one matching may be less straightforward if survey weights are involved. Finally, in the non-linear setting, the estimated effect of the covariate $x_i$ would depend on the value of the other covariates. We adopt the procedure in Stata described in Jann [38], which solves these problems in the following way:

- A hypothetical sample with replacement is drawn from both groups, whereby the probability of being selected from the sample is proportionate to the sampling weight. The number of observations *N* drawn from each sample is set to half of the total observations in both samples, such that $N = \dfrac{N^{t}+N^{t+1}}{2}$.
- To solve the problem of path dependence in the detailed decomposition, multiple estimations of health with a randomised order of the covariates are performed, and the obtained effects are averaged over all possible orderings [31]. The process is analogous to the approach described in Shorrocks [66].

We present descriptive and decomposition results for different population sub-groups by sex, age, education and income. In terms of age, we group individuals into four groups: youth (individuals of 16–24 years of age that are mostly in education), individuals that are at the first and second halves of their active labour market years (25–44 and 45–64 years of age, respectively), and individuals that are mostly out of the labour market (over 64 years of age). We use the relative income definition to divide the Irish population into three groups based on equivalised household disposable income: low-income individuals with less than 75% of the median income, middle-income individuals with incomes between 75 and 166% of median income, and high-income individuals with incomes above 166% of median income. While such income classification is arbitrary, it has been supported by previous studies on income deprivation (see for instance Whelan et al. [80]).

## Results

In this section, we present two sets of results. First, we review the main changes in socio-economic factors and self-assessed health during the first 5 years of the crisis in Ireland. Second, we present the decomposition results, which will shed light on the relative importance of changing demographic and socio-economic factors in driving changes in health observed between 2008 and 2013.

### Descriptive results

**Fig. 1:**
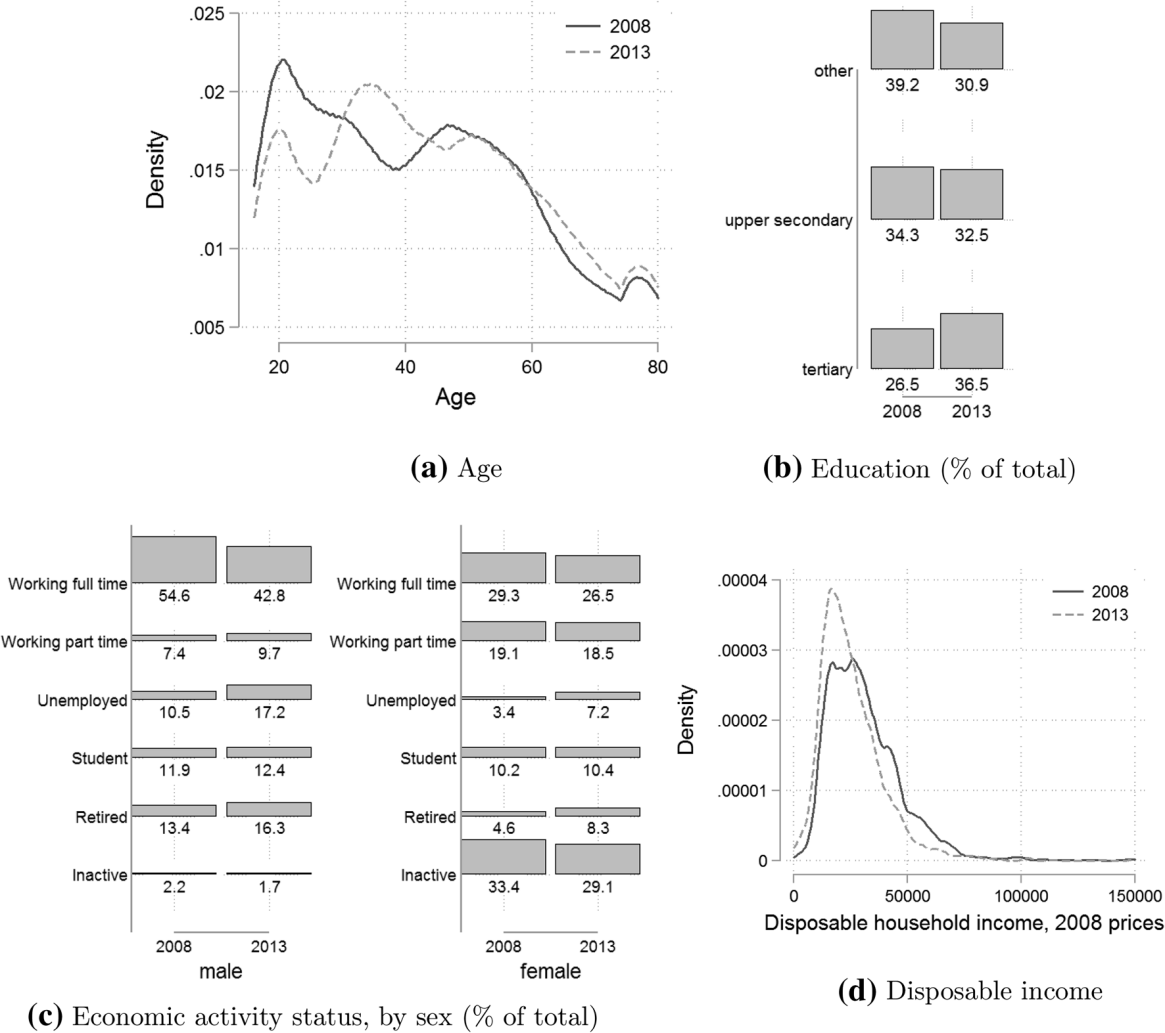
Distributional changes in the explanatory factors, 2008–2013

There are two potential drivers of change in population health over time. The first one is the compositional change in population factors that may be important for explaining health, such as age and income. The second one concerns the evolution of the health status of individuals from different socio-economic and demographic groups. Considerable changes in both demographic and socio-economic population characteristics occurred in Ireland between 2008 and 2013. First, a rapid population ageing could be observed. By 2013, the share of the population below 30 years of age had decreased significantly, while the share of population between 30 and 45 years and over 60 years of age had gone up (Fig. 1a). The observed trends are possibly driven by the decline in birth rates in the 1990s as well as intensified emigration. For example, the number of individuals annually leaving Ireland increased almost fourfold between 2008 and 2013, with net migration reaching a low point in 2013 and slowly starting to improve in 2014 [24]. Second, there has been an expansion of tertiary education and a related reduction in the share of the population with lower than upper secondary education (Fig. 1b). The observed increase in educational attainment may not only be a continuation of the trends in the years preceding the crisis (see for instance OECD [61]), but it may also partially reflect the decision of the youth to stay in education and delay their entry into the labour market until job prospects improve [33].

The economic recession was probably most strongly reflected by the labour market trends (Fig. 1c). Unemployment rate nearly doubled, with men affected less severely in relative terms than women (a change from 10.5 to 17.2% and from 3.4 to 7.2%, respectively). Men were more likely to increase part-time work than women. The share of students in the population has remained fairly stable, even though there was a decline in the fraction of the youth of up to 30 years of age. It is likely that some young individuals chose to stay in education longer, which would mean that the effect of the crisis on unemployment may be underestimated if this decision is not accounted for. In addition to this, we find a drop in inactivity levels and a rise in the rate of retirement. These trends most likely reflect cohort effects of increased female labour force participation over time. Finally, we observe a significant drop in income, with an increased concentration of individuals among lower income groups (Fig. 1d).

The crisis also brought about significant changes in financial strain and some environmental problems (Table 4). We find an increase in the composite index of financial strain by 76% and a small decline in the index of environmental issues. The share of individuals with arrears on hire purchase instalments or loan payments tripled in 5 years. Similar changes could be observed for the trends in arrears on utility bills and arrears on mortgage or rent payments. This trend is unsurprising given the crash of the housing market in Ireland, which reduced the property value of homeowners, many of whom had to keep paying their loans while faced with unemployment and reduced household income. In addition to this, nearly twice as many individuals reported having financial difficulties with paying their housing costs (90.5% increase), while the percentage of the Irish population reporting difficulties in making ends meet and a lack of capacity to face unexpected expenses rose by 61.9 and 40.5%, respectively. Contrary to our expectation, individuals from all income groups were affected by the increase in financial strain (Table 9 in the Annex). As shown elsewhere (see for instance a paper by Whelan et al. [80]), the relative rise in financial strain has been larger among higher income individuals, who were much less likely to experience financial strain before the crisis than lower income individuals. Finally, the population proportion experiencing environmental problems decreased by 15.4%. This change was driven in particular by the decline in noise and pollution, grime and other environmental problems, most likely brought about by decreased traffic due to the lower economic activity. However, we find that changes in factors comprising an index of environmental issues were not unidirectional. For example, there has been a significant increase in the share of the population reporting deteriorated housing quality, namely issues related to having damp and low light in the dwelling, while the share of the population reporting noise and pollution has decreased.

**Table 4:** Temporal changes in financial strain and environmental factors, 2008–2013

Variable	Mean ($\mu$)		Change (%)	Wald $\chi ^2$
	2008	2013		
Financial strain—composite	0.25	0.44	76.0	$p <0.000$
Arrears: mortgage/rent	0.04	0.10	150.0	$p < 0.000$
Arrears: utility bills	0.06	0.16	166.7	$p < 0.000$
Arrears: loans	0.02	0.06	200.0	$p < 0.000$
Fin. burden: housing cost	0.21	0.40	90.5	$p< 0.000$
Fin. burden: debts	0.11	0.15	36.4	$p<0.000$
Issues with: ends meet	0.21	0.34	61.9	$p <0.000$
Issues with: unexpected costs	0.37	0.52	40.5	$p < 0.000$
Environmental factors—composite	0.26	0.22	− 15.4	$p < 0.000$
Leaking roof, damp walls/floors	0.11	0.14	27.3	$p < 0.000$
Too dark in the dwelling	0.05	0.06	20.0	$p < 0.027$
Noise from neighbours/street	0.12	0.09	− 25.0	$p < 0.000$
Pollution, grime, other env. problems	0.08	0.04	− 50.0	$p < 0.000$
Crime violence or vandalism in the area	0.12	0.11	− 8.3	$p < 0.492$

Wald $\chi ^2$ statistics is calculated for difference in means

Finally, there has been a lot of heterogeneity in terms of changes in self-assessed health among population sub-groups (Table 5). Overall, the prevalence of poor self-assessed health rose from 13 to 15% between 2008 and 2013. In relative terms, the change has been more pronounced among younger, better educated and higher income individuals. In terms of age, individuals of 25–44 years of age were affected the most (33.3% increase in poor health), while there were no significant changes in health among other age groups. In terms of educational attainment, individuals with a tertiary degree were affected the most in relative terms (an increase in poor health by 80%), followed by individuals with an upper secondary degree (37.5% increase) and individuals with lower than an upper secondary degree (22.7% increase in poor health). In terms of income, the prevalence of poor health doubled from 4% to 8% for high-income individuals, followed by 27.3% increase for middle-income individuals. No significant change in health has been observed for low-income individuals. However, young individuals were significantly more likely to be in a low-income group in 2013 than in 2008, while the elderly population was less likely have low income (Fig. 3). Finally, the rate of decline in self-assessed health has been very similar among the full-time employees and the unemployed, which suggests that factors beyond economic activity status, such as increased psychosocial stress, may have been important in explaining changes in self-assessed health. In fact, we do not find significant changes in the logistic regression coefficients for poor self-assessed health after controlling for all of the explanatory factors (Fig. 4 in the Annex). This confirms our hypothesis that the change in population health was largely driven by compositional changes than return structure effects.

**Table 5:** Temporal changes in the prevalence of poor self-assessed health by population sub-groups, 2008–2013

Variable	Prevalence of poor health		Change (%)	Wald $\chi ^2$
	2008	2013		
Age groups				
16–24	0.04	0.04	0.0	$p < 0.360$
25–44	0.06	0.08	33.3	$p < 0.025$
45–64	0.15	0.17	13.3	$p < 0.117$
65 +	0.36	0.37	2.8	$p < 0.706$
Gender				
Male	0.11	0.13	18.2	$p < 0.003$
Female	0.14	0.16	14.3	$p < 0.020$
Education				
Upper to lower sec	0.22	0.27	22.7	$p < 0.001$
Upper secondary	0.08	0.11	37.5	$p < 0.001$
Tertiary	0.05	0.09	80.0	$p < 0.000$
Economic activity status				
Working full time	0.05	0.07	40.0	$p < 0.021$
Working part-time	0.11	0.09	− 18.2	$p < 0.233$
Unemployed	0.13	0.19	46.2	$p < 0.024$
Student	0.03	0.05	66.7	$p < 0.122$
Retired	0.34	0.34	0.0	$p < 0.965$
Inactive	0.26	0.28	7.7	$p < 0.539$
Income class				
Low income	0.20	0.21	5.0	$p < 0.783$
Middle income	0.11	0.14	27.3	$p < 0.000$
High income	0.04	0.08	100.0	$p < 0.001$
Poor self-assessed health	0.13	0.15	15.4	$p < 0.000$

Wald $\chi ^2$ statistics is calculated for difference in means

**Fig. 2:**
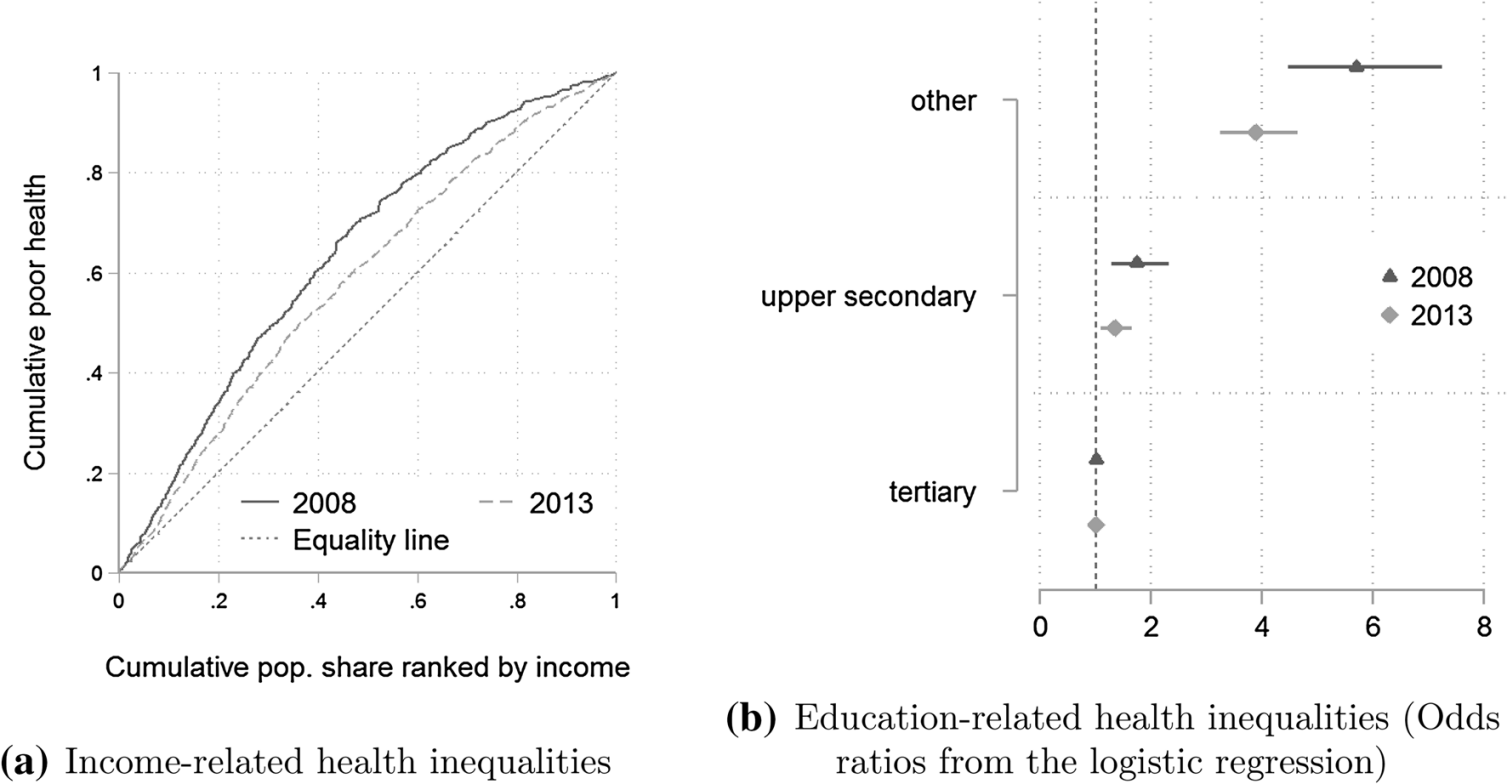
Changes in socio-economic health inequalities, 2008–2013

**Fig. 3:**
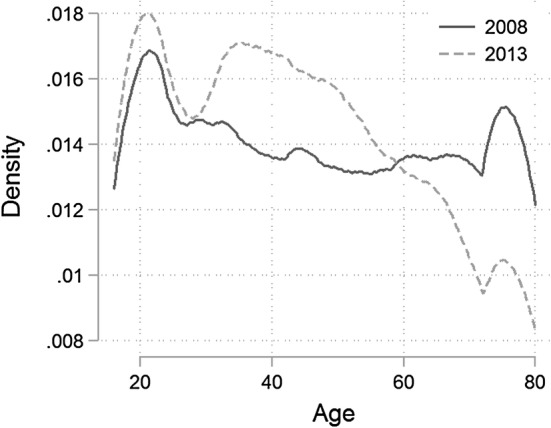
Changes in the age distribution among low-income individuals, 2008–2013

**Fig. 4:**
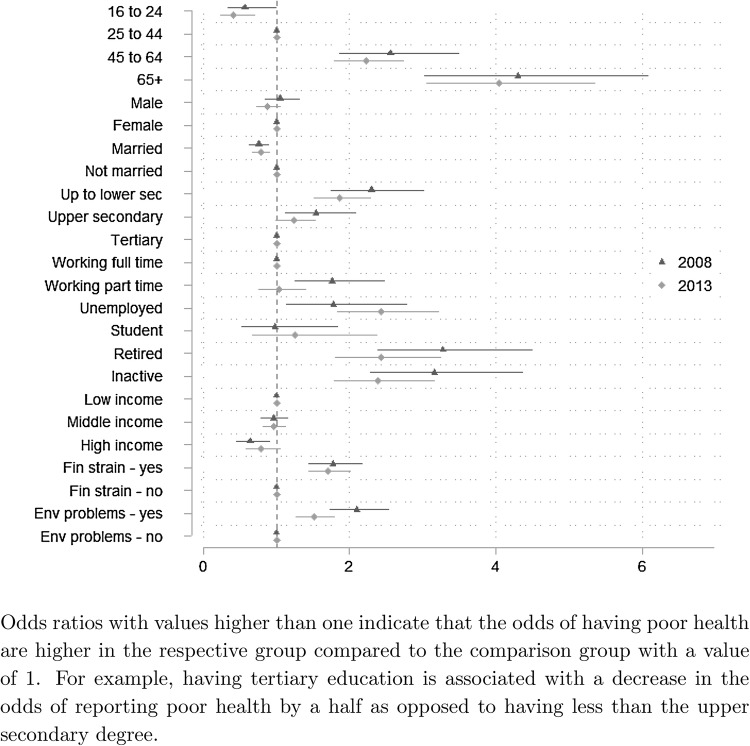
Adjusted odds ratios for reporting poor health before and after the economic crisis

The changes observed in the crisis period have had important implications for socio-economic health inequalities. For example, the larger relative increase in poor health among individuals with above-median income has had a reducing effect on income-related inequalities in self-assessed health. Perhaps the best way to summarise the change in income-related health inequalities is by comparing the concentration curves of both years (Fig. 2a). Similar to the Lorenz curve that is used to describe income inequality, concentration curve is a useful tool for examining inequalities in bi-variate distributions. The curve for the year 2013 lies visibly closer to the equality line, and the concentration index obtained from the curve for 2008 is almost twice as large compared to 2013 (− 0.28 and − 0.17, respectively). The observed change is statistically significant at 5% significance level. This suggests a marked decrease in income-related health inequalities. A similar conclusion can be obtained with respect to education-related health inequalities. The odds of having poor self-assessed health for someone with lower than upper secondary education compared to a person with tertiary education decreased from 6 to 4 (Fig. 2b).

The descriptive results so far suggest that the deterioration of self-assessed population health has been mirrored by unfavourable changes in demographic and socio-economic indicators, such as an ageing population, increased unemployment, reduced incomes and an increase in financial strain. However, some positive changes have occurred between 2008 and 2013 that may have had a counteractive effect on declining population health, such as the expansion of tertiary education and the decline in the prevalence of environmental issues. In addition to this, we find that the change in health has not been uniform across socio-economic groups, which led to a reduction in relative income- and education-related health inequalities.

### Decomposition results

In this section, we examine the drivers of change in the prevalence of poor self-assessed health in the Irish population and its sub-groups by age, sex, education and income. The top left panel of Table 6 shows the results for the Irish population as a whole, while the other columns present the results for different sub-groups. For the overall population, changes in socio-economic and demographic factors explained more than half of the change in the prevalence of poor self-assessed health. The rise in financial strain was the largest driver of the observed increase (1.41 p.p.), almost three times as large as the effect of changes in demographics (0.48 p.p.) and economic activity status (0.45 p.p.). The expansion of education, on the other hand, has had an opposite effect on health than the other factors, preventing a rise in the prevalence of poor self-assessed health by 0.51 p.p. After controlling for the increase in financial strain, income changes have shown no effect on changes in health. Changes in environmental factors (taken together) also did not contribute to explaining observed trends in health.

**Table 6:** Decomposition results for the Irish population and sub-groups by age, sex, education and income (2008–2013)

	All	By sex		By age			
	Population	Male	Female	16–24	25–44	45–64	65 +
Demographics	0.48***	0.66**	0.28	− 0.058	0.19*	− 0.039	− 0.36**
Education	− 0.51***	− 0.52***	− 0.60***	0.070	− 0.40*	− 0.83***	− 1.12***
Ec. activity status	0.45***	0.82***	0.26	0.12	0.12	0.44*	0.43
Income	0.10	0.060	0.15	− 0.28	0.27	0.090	− 0.052
Env. problems	− 0.11	− 0.057	− 0.18	0.047	− 0.11	− 0.16	0.088
Fin. strain	1.41***	1.15***	1.65***	0.77*	1.17***	2.62***	0.29
Total explained	1.816	2.118	1.564	0.660	1.249	2.122	− 0.732
Total change	2.349	2.513	2.176	0.909	2.033	2.030	0.664
*N*	17,955	8374	9581	2419	5413	5558	4565

	By education			By income			
	< Upper sec.	Upper sec.	Tertiary	Low	Middle	High	
Demographics	1.92***	0.61*	0.36	− 1.78***	1.69***	0.83**	
Education				− 0.68**	− 0.33***	− 0.59*	
Ec. activity status	1.04***	0.77**	0.56**	0.14	0.64***	0.37	
Income	− 0.041	− 0.15	0.37	− 0.51	0.055	0.039	
Env. problems	− 0.097	− 0.095	− 0.33	− 0.13	− 0.18	0.20	
Fin. strain	1.53***	1.45***	2.06***	2.30***	1.47***	0.0093	
Total explained	4.350	2.579	3.020	− 0.658	3.341	0.858	
Total change	4.500	3.204	3.814	0.352	3.400	3.407	
*N*	7200	5470	5285	6143	9244	2568	

Coefficients show the part of the change in poor self-assessed health (in p.p.) accounted for by different factors. Bootstrapped standard errors are calculated with 5000 replications: *$p < 0.05$, **$p < 0.01$, $p < 0.001$

Sub-group analysis by age, sex, education and income revealed non-uniform effects of different explanatory factors on health. First, we find differences with respect to age and sex. Interestingly, changes in economic activity status and demographic characteristics (mainly, age structure) contributed to the increased prevalence of poor health among males but not females, whereas increased financial strain contributed to a higher increase in poor health among females. In terms of age, the increased financial strain was important for explaining health decline among most age groups, in particular, individuals of 45–64 years of age (2.62 p.p.). On the other hand, the effect of the expansion of education on health was increasing with age, whereby individuals with 65 years and above experienced the largest protective effects of education on health. This result is due to a nearly linear increase in tertiary and secondary education attainment in the recent decades [62].

Second, the findings suggest varying effects of compositional changes in the examined factors on health by education and income. In terms of educational attainment, changes in demographic and economic activity status predicted changes in health better among the lower educated individuals, whereas increased financial strain was associated with a larger increase in the prevalence of poor self-assessed health among individuals with a tertiary degree. The effect of demographic changes on health varied between 1.92 p.p. for the least educated individuals and 0.36 p.p. for the most educated, the latter effect being insignificant at 5%. Variation in the effects of changes in economic activity status was somewhat smaller (from 1.04 to 0.56 p.p. for the least and most educated, respectively). The increased financial strain, on the other hand, had a higher effect on the change in health among individuals with a tertiary degree (2.06 p.p. compared to around 1.5 p.p. for the less educated). In terms of income, we find heterogeneous effects of changes in the demographic structure, economic activity status, and financial strain on health. Even though demographic changes (mainly, the change in the age distribution) contributed to worsened population health in the general population, they accounted for a 1.78 p.p. reduction in the prevalence of poor self-assessed health among low-income individuals. Changes in the economic activity status have had a significant contribution to increased prevalence of poor health of about 0.64 p.p. only among the middle-income group, while increased financial strain most severely affected the health of low- and middle-income individuals, but not individuals with high income. The effect of the increased financial strain on the rise in the prevalence of poor health was 2.3 p.p. and 1.47 p.p. for the low- and middle-income groups, respectively.

It is also informative to examine how changes in the variables comprising financial strain and environmental factors analysed separately contributed to the observed changes in health in the Irish population. Such exercise serves two purposes. First, it may shed some light on whether changes in objective (arrears) or subjective measures of financial strain were driving changes in health. Second, while the combined trends in the environmental factors taken together did not contribute to changes in health, we might find that changes in some of these factors taken alone did, as suggested by their trends depicted in Table 4 in the previous section. With respect to financial strain, we find that changes in both objective and subjective indicators had significant contributions to increased prevalence of poor self-assessed health (Table 7). The increased incapacity to face unexpected expenses was the largest driver of the observed increase in poor health (0.58 p.p.), even after accounting for all the other factors of financial strain. With respect to the environmental issues, changes in the variable summarising problems of leaking roof, damp walls or floors contributed to the increased prevalence of poor self-assessed health by 0.08 p.p., while changes in the noise from neighbours or the street contributed to the reduced prevalence of poor self-assessed health by 0.13 p.p. points after controlling for other environmental factors (Table 8). The effects of changes in pollution, grime, or other environmental problems were only significant when other environmental issues were not accounted for (0.16 p.p decrease in poor health). No effects of the change in darkness in the dwelling and change in crime, violence and vandalism on changes in health could be observed, as expected given only a minor change in the prevalence of these issues in the period of 2008–2013.

**Table 7:** Decomposition results for the Irish population using different indicators of financial strain (2008–2013)

Demographics	0.47***	0.43***	0.47***	0.50***	0.52***	0.50***	0.54***	0.50**
Education	− 0.61***	− 0.61***	− 0.61***	− 0.60***	− 0.60***	− 0.58***	− 0.50***	− 0.52***
Ec. activity status	0.57***	0.52***	0.54***	0.55***	0.58***	0.55***	0.53***	0.45***
Income	0.31**	0.28**	0.32***	0.27**	0.33***	0.25**	0.14	0.10
Env. problems	− 0.094	− 0.083	− 0.11	− 0.12	− 0.12	− 0.11	− 0.11	− 0.11
Arrears: mortgage/rent	0.29***							− 0.0053
Arrears: utility bills		0.67***						0.30*
Arrears: loans			0.36***					0.16*
Fin. burden: housing cost				0.81***				0.12
Fin. burden: debts					0.11**			0.031
Issues with: ends meet						0.64***		0.22
Issues with: unexpected costs							0.71***	0.58***
Total explained	0.939	1.211	0.969	1.417	0.816	1.241	1.314	1.816

Coefficients show the part of the change in poor self-assessed health (in p.p.) accounted for by different factors. Bootstrapped standard errors are calculated with 5000 replications: *$p < 0.05$, **$p < 0.01$, ***$p < 0.001$ (*N* = 17,955)

**Table 8:** Decomposition results for the Irish population using different indicators of environmental issues (2008–2013)

Demographics	0.47***	0.47***	0.49***	0.49***	0.51***	0.47***
Education	− 0.51***	− 0.50***	− 0.52***	− 0.50***	− 0.51***	− 0.51***
Ec. activity status	0.44***	0.44***	0.46***	0.45***	0.44***	0.44***
Income	0.081	0.086	0.092	0.093	0.10	0.10
Fin. strain	1.49***	1.58***	1.56***	1.59***	1.53***	1.41***
Environmental factors:						
Leaking roof, damp walls/floors	0.10***					0.081**
Too dark in the dwelling		− 0.0053				− 0.0022
Noise from neighbours/street			− 0.21***			− 0.13*
Pollution, grime, other env. problems				− 0.16**		− 0.044
Crime violence or vandalism in the area					− 0.019	− 0.011
Total explained	2.079	2.072	1.874	1.949	2.049	1.816

Coefficients show the part of the change in poor self-assessed health (in p.p.) accounted for by different factors. Bootstrapped standard errors are calculated with 5000 replications: $p < 0.05$, **$p < 0.01$, ***$p < 0.001$ (*N* = 17,955)

Overall, we find that an increase in financial strain between 2008 and 2013 has been the largest driver of the rise in the prevalence of poor self-assessed health in the general Irish population as well as among different population sub-groups. A smaller but significant effect was observed for the change in the economic activity status, whereas the expansion of tertiary education has had a protective effect on health. We also found non-uniform effects of compositional changes in explanatory factors across population sub-groups by age, sex, education, and income. We discuss some of the main results in the next section.

## Discussion

In this study, we examined the association between changes in socio-economic and demographic factors and changes in the prevalence of poor self-assessed health during the 2008 economic crisis in Ireland. We found that the increased financial strain has been the major driver of worsened population health. The effect became smaller but remained dominant even when the change in only one of the three objective measures of financial strain (arrears on mortgage payments, arrears on utility bills and arrears on loan payments) was taken into account. This result is in line with previous studies that have found a strong association between financial strain and health, in particular, mental health [35, 54]. Changes in the economic activity status were also significant predictors of worsened population health, while the effects of income change ceased to be significant after the increase in financial strain was accounted for. This result may suggest that changes in psychosocial rather than material factors induced by the 2008 economic crisis have been the major drivers of the increase in the prevalence of poor self-assessed health, such as increased stress related to the rise in job and home insecurity [51, 56].

Sub-group analysis provided us with further insights into the distinct pathways through which different socio-economic factors shaped the prevalence of poor health depending on age, education and income. First, our findings suggest that in relative terms, self-assessed health deteriorated the most among the individuals that were in the first half of their working age, including those that recently entered the labour market. High levels of unemployment at the beginning of one’s career might have profound scarring effects in the long run, meaning that the affected youth may be subject to more precarious job opportunities in the future, and as a result accumulated effects on health [33]. Health of the elderly, on the other hand, may be better protected from the changes in the economy due to retirement income and free access to medical services through the medical card.^5^ Second, the health of higher educated individuals was more severely affected by the crisis in relative terms compared to less educated individuals, which was surprising given that the effect of the changes in the economic activity status on health was found to decrease with education. There is evidence that better educated individuals tend to value their health in a more negative light than lower educated individuals [6]. It would be interesting to investigate whether better educated individuals are also more likely to be vulnerable to stress induced by increased financial strain and changes in the economic activity status and whether this may lead to education-related reporting differences in health during an economic crisis. In addition to this, we found that changes in financial strain affected the health of low- and middle-income individuals but not the high-income individuals, even though the high-income group experienced a higher relative increase in financial strain, as found elsewhere [79]. The result could be explained by the increased home insecurity and decreased health care affordability among the low-income individuals faced with financial strain, which may have psychosocial effects on health [56, 57]. Finally, the differential impact of the crisis on health via education and income has led to decreased socio-economic health inequalities in Ireland. Similar results have been found in some other European countries, such as Spain [22].

While our results are in line with a broad body of the literature, some potential limitations should be considered when interpreting the results. First, we use a subjective measure of health, which might be correlated to one’s general well-being. However, self-assessed health has been previously shown to be a good predictor of mortality and subsequent illness, and it is effective at capturing both mental and physical aspects of health [37, 40, 48]. Second, the model used in this paper does not allow for causal inference. Regardless of this, it remains informative in understanding the relative importance of changes in different socio-economic factors in shaping changes in health. In addition to this, the method used is path dependent, meaning that the interpretation depends on the assumptions of the relationship between health and socio-economic variables. We circumvent the problem by performing a large number of decompositions with randomised order and, therefore, obtain what can be considered an average contribution of each factor in shaping changes in health. Finally, the decomposition approach we employed in this paper was useful for looking at the changes in the covariates, but not for looking at their health returns. While the returns structure effects could be investigated using other parametric decomposition approaches, we do not expect to observe a significant contribution to the results because our logistic models have shown only small changes in the coefficients linking health to the studied socio-economic factors.

## Conclusion

In this paper, we set out to study the sources of change in population health in Ireland during the Great Recession (2008–2013). We examined the role of the changes in an array of socio-economic and demographic factors—population characteristics, education, economic activity status, income, financial strain, and environmental factors—on changes in the prevalence of poor self-assessed health. Our main hypothesis was that the increased financial strain would be an important driver of changes in population health. The results confirmed our hypothesis.

Overall, we find a 2.4 percentage point increase in the prevalence of poor self-assessed health in Ireland between 2008 and 2013. The increased financial strain has been associated with the largest increase in poor health in the general population as well as for different population sub-groups. We also find a somewhat smaller but significant effect of changes in the economic activity status on changes in the prevalence of poor self-assessed health. The increased levels of education have had a significant counterbalancing effect on health, preventing further increases in poor self-assessed health. Finally, there was a bigger increase in the prevalence of poor self-assessed health among individuals with higher income and higher educational attainment, which led to a reduction in socio-economic health inequalities.

A few important policy implications can be derived from our results. First, the expansion of educational attainment has shown significant protective effects on health. As many governments feel the pressure to tighten their public budgets (including education budgets) in the times of economic crisis, it is important to take into account the long-term effects of education on health when making investment decisions. Second, we find that the health impact of the 2008 crisis was non-uniform across different population sub-groups in Ireland, and the effect of different factors varied greatly depending on age, educational attainment and income. This finding calls for a targeted policy response that goes beyond income protection. For example, we find that the health of individuals in the first half of their working age was affected most severely in relative terms. This group is particularly vulnerable to experiencing further deterioration in health in long run due to scarring effects. Thus, policies that encourage this group to stay economically active could potentially be used to safeguard their health during an economic crisis.

## Annex

See Table 9, and Figs. 3 and 4.

**Table 9:** Temporal changes in the index of financial strain by population sub-groups, 2008–2013

Variable	Prevalence of fin. strain		Change (%)	Wald $\chi ^2$
	2008	2013		
Age groups				
16–24	0.31	0.57	83.9	$p<0.000$
25–44	0.28	0.48	71.4	$p<0.000$
45–64	0.23	0.43	87.0	$p<0.000$
65 +	0.14	0.23	64.3	$p<0.000$
Gender				
Male	0.24	0.43	79.2	$p<0.000$
Female	0.26	0.45	73.1	$p<0.000$
Education				
Upper to lower sec	0.33	0.49	48.5	$p<0.000$
Upper secondary	0.26	0.49	88.5	$p<0.000$
Tertiary	0.13	0.35	169.2	$p<0.000$
Economic activity status				
Working full time	0.19	0.35	84.2	$p<0.000$
Working part-time	0.26	0.46	76.9	$p<0.000$
Unemployed	0.57	0.70	22.8	$p<0.000$
Student	0.27	0.57	111.1	$p<0.000$
Retired	0.10	0.22	120.0	$p<0.000$
Inactive	0.33	0.48	45.5	$p<0.000$
Income class				
Low income	0.38	0.62	63.2	$p<0.000$
Middle income	0.23	0.42	82.6	$p<0.000$
High income	0.07	0.17	142.9	$p<0.000$

Wald $\chi ^2$ statistics is calculated for difference in means

## Footnotes

1 With an exception of persons living in institutions and collective households, as well as persons living in some offshore islands and small parts of national territory amounting to less than 2% of the population.

2 In EU-SILC, the age of individuals older than 80 years is coded as 80 instead of the actual age.

3 Labour market status = ‘disabled’ is excluded from the analysis due to extremely high odds of reporting poor health in this sample.

4 A comparison of the results based on the choice of the reference group has not shown significant differences, as expected given the similarities of the regression coefficients between years 2008 and 2013.

5 In 2011, around 91% of individuals in their seventies had access to medical cards, compared to less than 40% in the general population [28]. The latter observation could also explain why the effect of the increased financial strain on the health of the elderly has been negligible, while it was large and significant for working-age individuals.
